# Bardoxolone methyl blocks the efflux of Zn^2+^ by targeting hZnT1 to inhibit the proliferation and metastasis of cervical cancer

**DOI:** 10.1093/procel/pwaf044

**Published:** 2025-06-05

**Authors:** Yaxin Wang, Qinqin Liang, Shengjian Liang, Yuanyue Shan, Sai Shi, Xiaoyu Zhou, Ziyu Wang, Zhili Xu, Duanqing Pei, Mingfeng Zhang, Zhiyong Lou, Binghong Xu, Sheng Ye

**Affiliations:** School of Life Sciences, State Key Laboratory of Synthetic Biology, Tianjin University, Tianjin 300072, China; School of Life Sciences, State Key Laboratory of Synthetic Biology, Tianjin University, Tianjin 300072, China; MOE Key Laboratory of Protein Science, School of Medicine, Tsinghua University, Beijing 100084, China; Laboratory of Cell Fate Control, School of Life Sciences, Westlake University, Hangzhou 310024, China; School of Life Sciences, State Key Laboratory of Synthetic Biology, Tianjin University, Tianjin 300072, China; Department of Medical and Pharmaceutical Informatics, Hebei Medical University, Shijiazhuang 050017, China; School of Life Sciences, State Key Laboratory of Synthetic Biology, Tianjin University, Tianjin 300072, China; School of Life Sciences, State Key Laboratory of Synthetic Biology, Tianjin University, Tianjin 300072, China; Division of Life Sciences and Medicine, University of Science and Technology of China, Hefei 230026, China; Laboratory of Cell Fate Control, School of Life Sciences, Westlake University, Hangzhou 310024, China; Laboratory of Cell Fate Control, School of Life Sciences, Westlake University, Hangzhou 310024, China; MOE Key Laboratory of Protein Science, School of Medicine, Tsinghua University, Beijing 100084, China; School of Life Sciences, State Key Laboratory of Synthetic Biology, Tianjin University, Tianjin 300072, China; School of Life Sciences, State Key Laboratory of Synthetic Biology, Tianjin University, Tianjin 300072, China

## Dear Editor,

Cervical cancer is one of the common malignant neoplasms of the female reproductive system, particularly in low-income and middle-income countries globally, resulting in over 660,000 women diagnosed, and over 348,000 deaths annually (Abu-Rustum et al., 2023; Sahasrabuddhe, 2024). Growing evidence indicates that the human Zn^2+^ transporter hZnT1 expression is significantly higher in cervical carcinoma and is positively correlated with lymph node metastasis or distant metastasis in this cancer (Zhang et al., 2022). HZnT1 enhances the metastatic ability and proliferative capacity of cervical cancer cells by influencing immune factors and cancer-related signaling pathways like MAPK, Ras-ERK, and Wnt (Beharier et al., 2012; Zhao et al., 2016). Worse still, the up-regulation of hZnT1 is resistant to anti-cancer drugs, such as vinvristie, paclitaxel, and gemcitabine (Zhang et al., 2022). Among the ten hZnT members, hZnT1 is the only Zn^2+^ transporter predominantly located on the plasma membrane, mediating Zn^2+^ efflux from the cytosol to extracellular environments, to protect cells against Zn^2+^ toxicity (Chen et al., 2024; Long et al., 2024; Stiles et al., 2024; Sun et al., 2024). Therefore, deciphering the physiological function of hZnT1 and developing therapeutic agents that specifically target this transporter could be an effective strategy for cervical cancer treatment.

**Figure 1.**
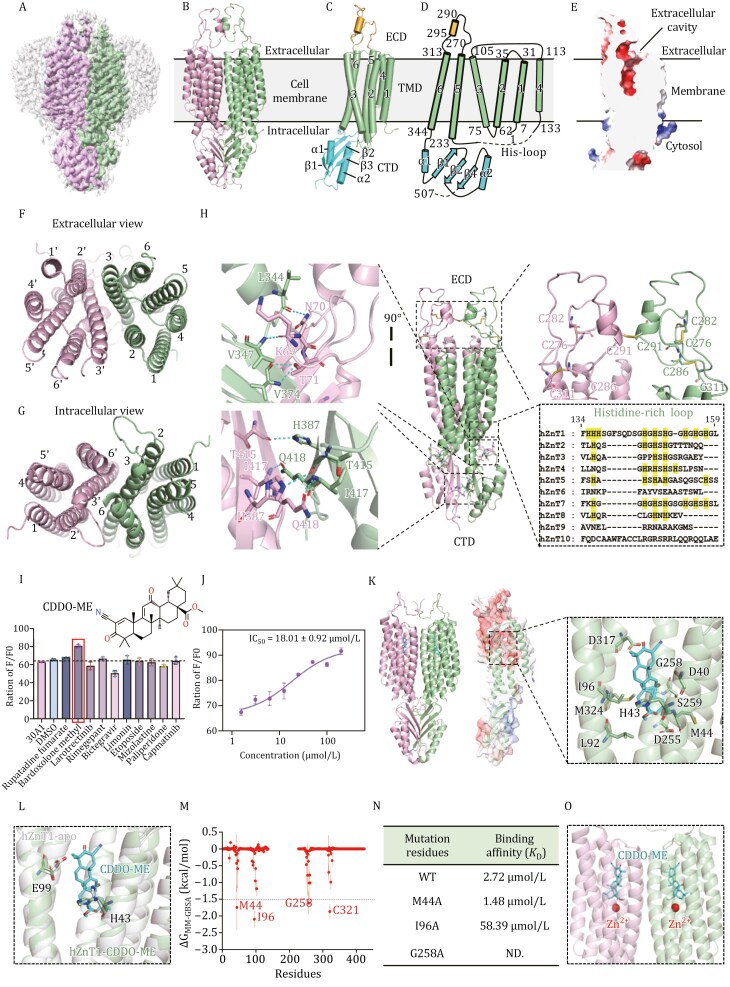
**Structure-based inhibitor discovery of hZnT1.** (A) The EM density of hZnT1 at 3.0 Å resolution. Densities corresponding to the two hZnT1 protomers are colored green and pink, respectively. The surrounding micelle is shown in light gray. (B) Cartoon representation of the dimeric hZnT1 structure. (C) The side view of the hZnT1 protomer structure, which is organized into the ECD, TMD, and CTD. (D) The topological diagram of hZnT1. The starting and ending residue numbers of helices and β-sheets are labeled aside. (E) Coronal section of the electrostatic potential surface map of the hZnT1 protomer. Surface colors indicate Coulombic potentials (red, negative; white, neutral; blue, positive). The extracellular cavity of hZnT1 OF form is labeled. (F and G) The extracellular view and intracellular view of the hZnT1 TMD. (H) Overview of the ECD and CTD of hZnT1. Residues in the dimer interface forming hydrogen bonds are shown in sticks. The histidines in His-rich loop region are colored in yellow. (I) Preliminary inhibitory effects of candidate inhibitors on hZnT1 and the chemical formula of Bardoxolone methyl (CDDO-ME). (J) The IC_50_ of CDDO-ME on Zn^2+^ transport assay. (K) The overall structure of hZnT1-CDDO-ME. The CDDO-ME binds in the Zn^2+^ bound cavity and interacts with the residues on TM regions. (L) The conformational changes of H43 and E99 in apo and CDDO-ME bound hZnT1. (M) The crucial four residues (M44, I96, G258 and C321) contribute the most to the free energy. (N) The binding affinities of CDDO-ME and WT or mutated hZnT1. (O) The mechanism of CDDO-ME inhibiting hZnT1 transport of Zn^2+^. The CDDO-ME is located on top of Zn^2+^ and blocks the efflux channel of Zn^2+^ from cytoplasm to extracellular milieu.

To develop inhibitors targeting hZnT1, we determined the cryo-electron microscopy (cryo-EM) structure of hZnT1 at a resolution of 3.0 Å (PDB: 9KZW), which possesses a homodimer in an outward-facing (OF) conformation (Figs. 1A and S1; Table S1). The overall structure of hZnT1 adopts a “*mushroom*”-shaped homodimeric architecture with the transmembrane domain (TMD) and cytosolic domain (CTD) of two protomers tightly packed (Fig. 1B). Each hZnT1 protomer comprises three parts: an extracellular domain (ECD), a TMD and a cytosolic CTD (Fig. 1C). The first region ECD (residues 271–312) is formed by the linker between TM5 and TM6, mediates intersubunit dimerization through hydrophobic interactions, a feature not observed in other ZnT family members (Bui et al., 2023; Lu et al., 2009; Xue et al., 2020). The distinctive extracellular cysteine-rich ECD in hZnT1 formed an inter-subunit disulfide bond (C291–C291) and two intra-subunit disulfide bonds (C276–C282, C286–C311) (Fig. 1D and 1H). This unique arrangement is primarily responsible for sequestering extracellular Cu^2+^ and facilitating their efficient transport, while exhibiting minimal impact on Zn^2+^ transport, which expands the extra physiological function of hZnT1 beyond its role as Zn^2+^/H^+^ and Zn^2+^/Ca^2+^ exchanger (Gottesman et al., 2022; Li et al., 2024; Shusterman et al., 2014). The second part TMD of each hZnT1 protomer contains six transmembrane helices (TM1–6), forming a Zn^2+^ transport pathway (Fig. 1D, 1F, and 1G). Specially, residues of TM2 and TM3 interdigitate at the dimer interface and establish a complex network of hydrophobic interactions to promote the dimer formation in the TMD (Fig. S2). The transmembrane helices TM2, TM3, TM5 and TM6 are tightly bundled at the cytosolic half of the TMD in both protomers, and collectively create a cavity that is solvent accessible from the extracellular milieu but entirely sealed from the cytoplasmic side, indicating that our hZnT1 structure is in an OF state (Fig. 1E). Additionally, a His-rich loop flanked by TM4 and TM5 is located on the cytosolic side, with the majority invisible due to its flexible nature, except residues 134–136 near the C-terminal end of TM4 and residues 228–232 near the N-terminal end of TM5 build according to the map density (Figs. 1H and S5). The last region CTD (residues 345–507) is composed of two short α helices (α1–2) and three paralleled β sheets (β1–3), contributing the extensive interactions to stabilize the dimeric state of hZnT1 (Fig. 1D). Exquisitely, the β sheets of one protomer tightly pack against that of another protomer through hydrophobic interactions and four hydrogen bonds between H387–T415 and I417–Q418. In addition, K69, N70, and T71 in the extended loop between TM2–TM3 protrude into the neighboring subunit and tuck into the pocket between β1 and the loop of TM6-α1, establishing pivotal hydrogen bonds with L344, V347, and V374 to further strengthen the stability of hZnT1 homodimer (Fig. 1H and Table S2).

With the detailed structural and functional insights, we initiated a virtual screening based on a marketed drug library to explore the potential hZnT1 inhibitors as a strategy for the treatment of cervical cancer. This screening yielded ten candidate compounds with high binding affinity to hZnT1, exceeding −10 kcal/mol (Fig. 1I and Table S3). Followingly, we evaluated the inhibitory activity of these candidates on hZnT1 utilizing a FluoZin-3 fluorescence assay in HEK-293F cells (Gottesman et al., 2022). Among them, the compound Bardoxolone methyl (CDDO-ME) demonstrated the strongest inhibitory potency, effectively decelerating the hZnT1 transport activity with a half maximal inhibitory concentration (IC_50_) of 18.01 ± 0.92 µmol/L (Fig. 1I and 1J). To further substantiate that CDDO-ME specifically targets hZnT1, we employed the biolayer interferometry (BLI) assay to determine the binding affinity between the two entities. The binding affinity of CDDO-ME and hZnT1 is 2.72 µmol/L, indicating a strong interaction (Figs. 1N and S7A).

Since CDDO-ME really targets hZnT1, what is the precise inhibitory molecular mechanism of this compound? To investigate this issue, we solved the cryo-EM structure of hZnT1 in complex with CDDO-ME at a resolution of 3.78 Å (PDB: 9L00) (Figs. 1K, S3 and S4; Table S1). The structure revealed that CDDO-ME exquisitely extends into the Zn^2+^-bound cavity primarily through hydrophobic interactions, aligning parallel to the transmembrane helices (Figs. 1K and S6B). Superimposing the apo and CDDO-ME bound hZnT1 structures illustrated a noticeable significant movement of H43 and E99, which also substantiates that these conformational changes are indeed attributable to the binding of the inhibitor (Figs. 1L and S6A).

To reveal the interaction mode between CDDO-ME and hZnT1, we performed conventional molecular dynamic (CMD) simulations, constructing a complex simulation system, including hZnT1, CDDO-ME, water, KCl, and POPC (Fig. S6C). The stability of hZnT1 within this system was evaluated through three long-time simulations. The root mean square fluctuation (RMSF) and root mean square deviation (RMSD) of hZnT1 manifested that the hZnT1 is generally stable, with an RMSD distributed within 4 Å, an RMSF distribution within 2 Å, and an overall radius of gyration at 31–32 Å (Fig. S6D–F). Then, the binding free energy calculation and decomposition were carried out using the molecular mechanics generalized born surface area (MMGBSA) method. The binding stability of the CDDO-ME was assessed, with RMSD stabilizing at 0.5 Å (Fig. S6G). The ΔG_MMGBSA_ for the binding of CDDO-ME to hZnT1 was −53.19 kcal/mol, with residues M44, I96, G258, and C321 contributing most significantly to the free energy (Fig. 1M). Upon mutating these three residues overlapped with cryo-EM hZnT1-CDDO-ME structure interactions, the binding affinity of I96A significantly attenuated to 58.39 µmol/L, and even worse, the mutation of G258A directly led to the affinity to be completely undetectable, highlighting their pivotal roles in the binding of CDDO-ME (Figs. 1N and S7). Furthermore, we conducted a comparative analysis of the binding sites of CDDO-ME and Zn^2+^ within the transmembrane domain of hZnT1 to ascertain whether they exhibit any spatial overlap. The results revealed that CDDO-ME is located on the top of Zn^2+^, blocking the efflux channel of Zn^2+^ from cytoplasm to extracellular milieu (Fig. 1O). Structural superimposition of the Zn^2+^ unbound OF hZnT1 homodimer, Zn^2+^ bound inward-facing (IF) hZnT1 (PDB: 8XMF) (Long et al., 2024) and CDDO-ME-hZnT1 further elucidated the inhibitory mechanism of CDDO-ME. Comparative structural analysis revealed that hZnT1 experiences comparable rearrangements in the TMs during Zn^2+^ transport, with TM3 and TM6 remaining relatively stationary, while the other four TMs undergo significant rotations to facilitate exposure of the Zn^2+^ binding site to either the extracellular milieu or the cytoplasm (Fig. S8A, S8B, S8D, and S8E). Critically, the CDDO-ME occupies the Zn^2+^-transport cavity and sterically hinders the movement of TM2, thereby preventing the conformational transition of hZnT1 from an OF to IF state (Fig. S8C).

**Figure 2.**
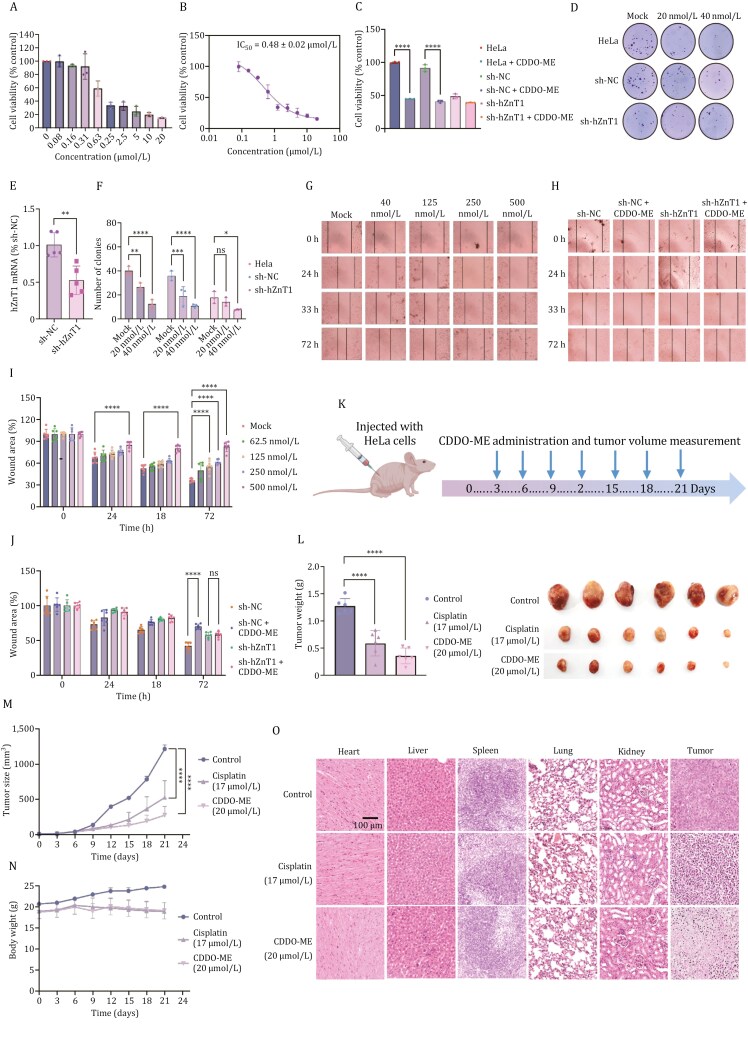
**CDDO-ME inhibits the proliferation and metastasis of cervical cancer cells *in vitro* and *in vivo*.** (A) The HeLa cell viabilities were detected using CCK-8 after different concentrations of CDDO-ME treated. (B) The IC_50_ of CDDO-ME on HeLa cells. (C) The viabilities of HeLa sh-NC and sh-hZnT1 cells were tested after 0.5 µmol/L of CDDO-ME treated. (D) The colony formations of 20 nmol/L and 40 nmol/L CDDO-ME on HeLa, sh-NC, and sh-hZnT1 cells. (E) The sh-hZnT1 of HeLa cells. (F) Statistical results of (D). (G) Representative images of different concentrations of CDDO-ME inhibited the migration of HeLa cells. The scale length represents 100 µm. (H) Representative images of sh-NC, sh-hZnT1 combined with 0.25 μmol/L CDDO-ME inhibited the migration of HeLa cells. The scale length represents 100 µm. (I) Statistical results of (G). (J) Statistical results of (H). (K) The schematic diagram of the experimental protocol. The xenograft model mice were inoculated with 5 × 10^6^ HeLa cells for 13 d, and drugs were injected every 3 days. (L) The tumor volume and weight in different groups (*n* = 6). (M) The tumor volume growth curve in different groups (*n* = 6). (N) The body weight changes curve in different groups (*n* = 6). (O) Representative tissue sections of major organs and tumor tissues in different administration groups. All the experiments were repeated three times. Data are mean ± SEM. Significances were determined using one-way analysis of variance (ANOVA) with Dunnett’s multiple comparisons test. *, *P* < 0.05; **, *P* < 0.01; ***, *P* < 0.001; ****, *P* < 0.0001; ns, not significant.

To dissect the CDDO-ME mediated inhibition of hZnT1 on cell proliferation, the HeLa cell viabilities were detected utilizing cell counting kit-8 (CCK-8) and colony formation assays. The result of CCK-8 assay manifested that the inhibitory effect of CDDO-ME on HeLa cells viability increased with concentration, with an IC_50_ value of 0.48 ± 0.02 µmol/L (Fig. 2A and 2B). Colony formation experiments further verified the inhibitory activity of CDDO-ME on the proliferation of single HeLa cell, showing a significant reduction in the number of colonies at 20 nmol/L or 40 nmol/L CDDO-ME (Fig. 2C and 2D). Knocking down the endogenous hZnT1 in HeLa cells by shRNA also significantly reduced the cell viability and number of colonies, suggesting that CDDO-ME inhibits the proliferation of HeLa cells by specifically targeting hZnT1 (Fig. 2D–F).

Given the highly metastatic nature of tumor cells, we performed the wound healing experiment to evaluate the effect of CDDO-ME on the migration of HeLa cells. The HeLa cells were incubated with gradually increasing concentrations of CDDO-ME, and the cell wound areas were measured every 24 h. As shown in Fig. 2G and 2I, the mock group untreated with CDDO-ME had the fastest migration, leaving only approximately 30% of the wound area after 72 h. In contrast, the wound area of 500 nmol/L CDDO-ME group occupied more than 80% of the initial total area after 72 h. Additionally, the migration rate of HeLa cells was apparently reduced after shRNA-hZnT1 transfection. On this basis, adding 250 nmol/L CDDO-ME did not further inhibit cells migration, proving that hZnT1 is the target of CDDO-ME in inhibiting HeLa cells metastasis (Fig. 2H–J).

Female nude mice were utilized to establish the HeLa tumor xenograft model to investigate the inhibitory effects of CDDO-ME on the growth of cervical carcinoma *in vivo*. Mice were administered with CDDO-ME or cisplatin intraperitoneally every three days (Fig. 2K). The results indicated that both the cisplatin and the CDDO-ME-treated groups exhibit remarkable inhibitory effect in the growth of cervical carcinoma tumors. Tumor volume growth over time revealed a continuous increase in the control group, whereas the administration group exhibited varying degrees of growth inhibition. Remarkably, tumor volumes treated with 10 mg/kg (20 µmol/L) CDDO-ME were notably smaller in comparison to those treated with 5 mg/kg (17 µmol/L) cisplatin, indicating a potentially superior therapeutic efficacy (Fig. 2M). Simultaneously, the body weights of mice in both cisplatin and CDDO-ME treated groups did not significantly differ from the control group, suggesting that CDDO-ME does not adversely affect normal metabolism and exhibits low toxic side effects (Fig. 2N). After seven administrations, mice were euthanized and tumors were subsequently dissected. The results showed that the tumor weights in both cisplatin and CDDO-ME treatment groups were markedly lower than that of the control group, with the 20 µmol/L CDDO-ME dosage achieving a tumor weight inhibition rate exceeding 72% (Fig. 2L). To further explore the pharmacological mechanism of CDDO-ME *in vivo*, frozen sections and hematoxylin and eosin (H&E) staining were performed on the tumor tissues and various organs. In comparison to the control group, all treatment groups exhibited varying degrees of tissue damage, with the 20 µmol/L CDDO-ME group demonstrating the most pronounced nuclear dissociation and tissue necrosis. Moreover, H&E staining of major organs (heart, liver, spleen, lung, and kidney) revealed no obvious damage, further confirming the exceptional biological safety profile of CDDO-ME (Fig. 2O).

In conclusion, this study presents, for the first time, a report on the remarkable potential of CDDO-ME in the treatment of cervical cancer for the first time. All the assays employed on cells and *in vivo* consistently demonstrate that CDDO-ME exhibits potent inhibitory effects on the proliferation and metastasis of cervical cancer cells. We also clarify its molecular mechanism by blocking the hZnT1’s transport of Zn^2+^. These findings endow hZnT1 as a promising target for the development of cervical cancer drugs and expand the scope of CDDO-ME’s application in tumor therapy. Also, the elucidation of hZnT1-CDDO-ME complex structure and the pivotal residues mediating CDDO-ME binding paves the way for the future optimization of more potent CDDO-ME derivatives.

## Supplementary Material

pwaf044_Supplementary_Materials

## Footnotes

We thank the Westlake University Biomedical Research Core Facility and Electron Microscopy Facilities of Zhengzhou University for Cryo-EM data collection. This work was supported by Ministry of Science and Technology (2024YFC3407300 to Y.W., 2024YFA0916800 to B.X., and 2020YFA0908500 to S.Y.), the National Natural Science Foundation of China (31971127 to S.Y., 81801998 to Y.W., and 82404583 to S.S.).

The authors declare no competing interests and their agreement to publish. All of them confirm their willingness to participate. All institutional and national guidelines for the care were followed.

Y.W., B.X., and Q.L. designed the studies, analyzed the data; S.L., Z.X., M.Z., D.P., and Y.S. collected the cryo-EM data and determined the structures; B.X., Q.L., S.S., Z.W., and Y.Z. performed the experiments; Y.W., Z.L., and S.Y. conceived and supervised the project and prepared the manuscript.

All animal studies and experimental procedures were approved by the Animal Care and Use Committee of the animal facility at Tianjin University.

All data are available in the main text or the Supplementary materials.
