# Supplementary material for: Bardoxolone methyl blocks the efflux of Zn2+ by targeting hZnT1 to inhibit the proliferation and metastasis of cervical cancer
Source: Protein Cell. 2025 Jun 5;16(11):991–6. doi: 10.1093/procel/pwaf044 (PMC12698182; doi:10.1093/procel/pwaf044)
Supplement: pwaf044_Supplementary_Materials [file pwaf044_supplementary_materials.pdf]

Supplementary Materials

Materials and Methods

Cells lines

HEK-293T, HEK-293F, Sf9 and Hela cells were purchased from ATCC. The HEK-293T and Hela cells were cultured in Dulbecco's modified Eagle's medium (DMEM) containing 10% fetal bovine serum (FBS), 100 U/ml penicillin, and 100 mg/ml streptomycin (Gibco, USA) at 37 °C with 5% CO_2 in a humidified incubator. The HEK-293F were cultured in culture medium SMM 293-TI (Sino Biological, China) containing 10% FBS, 100 U/ml penicillin, and 100 mg/ml streptomycin (Gibco, USA) at 37 °C, 5% CO_2, 220 rpm in a shaker. The Sf9 cells were cultured in SIM SF medium (Sino Biological, China) at 28°C.

Expression and purification of wild type and mutated hZnT1

The full-length hZnT1 gene containing a C-terminal TwinStrep tag was cloned into a pEG-BacMam vector and the cloned vectors were transformed into DH10Bac competent cells. The extracted bacmids were transfected sf9 cells using X-tremeGENE 9 DNA Transfection reagent (Roche, Switzerland). The low-titer viruses were harvested after 4 days, and then amplified to generate high-titer virus stock. The viruses were used to infect HEK-293F cells at a multiplicity of infection (MOI) of 10, supplemented with 10 mM sodium butyrate to boost protein expression.

HEK-293F cells were cultured in suspension at 37 °C for 48 h and harvested by centrifugation at 3,000 g. The cell pellets were re-suspended in buffer A (25 mM Tris-HCl, 150 mM NaCl, pH 8.0) supplemented with a protease inhibitor cocktail (1 μg/ mL pepstatin, 1 μg/ mL leupeptin, 1 μg/ mL aprotinin and 1 mM PMSF) and homogenized by sonication on ice.

hZnT1 was extracted with 1.5% (*w/v*) n-Dodecyl-β-D-Maltopyranoside (DDM) (Anatrace, USA) by gentle agitation for 2.5 h on ice. After extraction, the supernatants

were collected by centrifugated at 48,000 g for 40 min, and incubated with Strep-Tactin Sepharose resins (IBA, Germany). After 1 h, the resins were collected on a disposable gravity column (Bio-Rad, USA), washed with buffer B (buffer A + 0.05 % DDM) and eluted with 10 mM desthiobiotin. The protein samples were further purified on a Superose 6 increase 10/300 GL column (GE Heathcare, USA) with buffer C (buffer A + 0.02 % DDM). The proteins were collected and analyzed by SDS-PAGE to over 95% purity, and then concentrated to 10 mg/ml for cryo-EM data collection.

Cryo-EM data collection

For cryo-EM sample preparation, the purified hZnT1 and hZnT1-CDDO-ME proteins at 10 mg/ml were applied to a glow-discharged holey carbon grids (Quantifoil Au R1.2/1.3, 300 mesh), blotted under 100% humidity at 8°C and plunged into liquid ethane using a Mark IV Vitrobot (Thermo Fisher Scientific, USA). Micrographs of hZnT1 were acquired on a Titan Krios electron microscope (FEI) operating at 300 kV, equipped with the GIF-Quantum energy filter and a K3 Summit direct electron detector (Gatan). SerialEM software (FEI) was used for automated data collection following standard FEI procedure. Images were recorded at a normal magnification of 270,000×, corresponding to a pixel size of 0.45 Å per pixel and with a set defocus range of -0.5 to -1.2 μm. Each micrograph was dose-fractionated to 32 frames recorded every 0.075 s under a dose rate of 4.2 e-/pixel/s, resulting in an accumulated dose of ~50 e^{-}/Å^{2}. Cryo-EM data of hZnT1-CDDO-ME were collected with a 300 kV Titan Krios electron microscope (Thermo Fisher Scientific, USA) with a Falcon 4 direct electron detector. Images for hZnT1-CDDO-ME were recorded at 96,000× magnification and calibrated at a super-resolution pixel size of 0.83 Å/pixel. The exposure time was set to 5.58 s with a total accumulated dose of 50 electrons per Å^{2}. A total of 12941 micrographs were automatically recorded using EPU and were collected with a defocus range from -2.0 μm to -1.0 μm.

Image processing and model building

A flowchart for the hZnT1 data processing using cryoSPARC suite is presented in

Fig. S1. All dose-fractioned images were motion-corrected and dose-weighted by patch motion correction and the contrast transfer function (CTF) of each micrograph was estimated by patch CTF estimation in cryoSPARC(Rohou and Grigorieff, 2015). The initial particles of hZnT1 were picked from a few micrographs using blob picker in cryoSPARC V4 and 2D averages were generated(Punjani et al., 2017). Final particle picking was done by template picker using templates from those 2D results. After three rounds of 2D classification, ab-initio reconstruction, non-uniform refinement and local refinement for reconstructing the density map. For hZnT1-CDDO-ME, a total of 12,090 micrographs were manually selected for further processing. All micrographs were selected to perform blob picker and 2D classification. Particles from high-quality 2D classes were then used for training in Topaz. After Topaz extraction and multi-rounds of 2D classification, ab-initio reconstruction and heterogeneous refinement, a total of 350,390 particles (160 pix, bin2) were selected for three classes in ab initio reconstruction, and the best class was selected for non-uniform refinement in the C2 symmetry. To further improve the resolution, particle subtraction and local refinement were performed on 142,341 particles (320 pixels, bin1). Last, a map with an overall resolution of 3.78 Å with 142,341 particles was achieved. All the maps were low-pass filtered to the map-model FSC value. The reported resolutions were based on the FSC=0.143 criterion. The initial model of hZnT1 was generated by hZnT8 (6XPE)(Xue et al., 2020) and the initial model of hZnT1-CDDO-ME was generated by hZnT1 (8XMA)(Long et al., 2024). Then, we manually completed and refined the model using Coot(Emsley et al., 2010). Subsequently, the models were refined against the corresponding maps by PHENIX(Adams et al., 2010). The statistics for the models' geometries were generated using MolProbity(Chen et al., 2010) (Table S1). All the figures were prepared in PyMol and Chimera(Pettersen et al., 2004).

HEK-293F cell based Zn^{2+} transport assay

HEK-293F cells expressed hZnT1 were harvested 36 h after virus infection. To remove residual culture medium and Zn^{2+}, the cells were centrifuged and washed with

uptake buffer (20 mM Hepes, 125 mM KCl, 5 mM NaCl, 1.8 mM $CaCl_2$, 10 mM Glucose, 10 μM Phenanthroline, pH 7.4) three times, and then re-suspended in uptake buffer to a final concentration of 1.5 × 10^6 cells/mL. To measure Zn^{2+} uptake, 100 μL of cell suspensions were added into a 96-well plate (Corning, USA) and then added 1 μM FluoZin-3 (Thermo, USA), 0.01% digitonin, and 9 μM $ZnCl_2$. The FluoZin-3 fluorescence was monitored every 15 s using a Microplate Reader at the excitation wavelength of 490 nm and emission wavelength of 525 nm. In order to ascertain the rate of Zn^{2+} uptake, the linear phase of the uptake measurement which represents the response subsequent to the addition of zinc, was determined by fitting the data to a linear regression equation in OriginPro 8. The slope of the fit represented the rate of Zn^{2+} uptake.

Virtual screening

The cryo-EM structure of hZnT1 was used for virtual screening. Marketed drugs (~2300 compounds) were screened using the molecular docking program Vina(Trott and Olson, 2010). A semi-flexible docking protocol was used to perform the calculations, and the size of the binding pocket search space was 30 × 20 × 24 $Å^3$. The global search exhaustiveness value was set to 50. The maximum energy difference between the optimal binding mode and the worst case was set to 5 kcal/mol.

Preliminary inhibitory effects of candidate inhibitors

The inhibitory activities of candidate compounds against hZnT1 were evaluated based on the Zn^{2+} transport assays. 100 μl of HEK-293F cells were seeded to a 96-well plate, and then the different concentrations of candidate inhibitors or DMSO were added and incubated on ice for 1 h. The following reagents were added sequentially: 1 μM FluoZin-3, 0.01% digitonin, and 9 μM $ZnCl_2$. Zn^{2+} uptake level was monitored for 10 min using a Microplate Reader (Tecan, Austria). The inhibitory efficacies were assessed via a reduction in Zn^{2+} uptake activity compared to the control cultures. EC_{50} was calculated using GraphPad Prism 9.

Affinity determination by Bio-Layer Interferometry (BLI)

Affinity assays were performed on an Octet® R8 biolayer interferometry instrument (Sartorius, Germany) at 25 °C with shaking at 1,000 rpm. To measure the affinity of hZnT1 with CDDO-ME, Super Streptavidin (SSA) biosensors (Sartorius, Germany) were hydrated in water for 30 min prior to 60 s (sec) incubation in a kinetic buffer (0.1 M Hepes,1.5 M NaCl, 0.03 M EDTA, 0.5% (*v/v*) Surfactant P20, pH 7.4). The purified hZnT1 were loaded in a kinetic buffer for 120 s prior to baseline equilibration for 120 s in a kinetic buffer. The data were baseline subtracted before fitting was performed using a 1:1 binding model and the Octet® R8 data analysis software. K_D, K_a, and K_d values were evaluated with a global fit applied to all data.

MD simulations

The simulation system containing hZnT1, POPC, and CDDO-ME was generated by CHARMM-GUI (box size 95Å × 93Å × 152Å). MD simulations were performed using AMBER22(D.A. Case, 2022). The Amber ff19SB force field, the lipid21 force field and the GAFF force field were applied to the protein, POPC bilayer and CDDO-ME, respectively. TIP3P water model and Cl^- were added to solvate and neutralize the membrane-protein systems, and added Joung/Cheatham ion parameters(Cheatham and Joung, 2009). The solvated system totally contained 136774 atoms. The other parameters were the same as we set before(Shi et al., 2023). Finally, the time step for all MD simulations was set to 2 fs and MD was performed for 500 ns with C_α constrained (1 kcal mol^{-1} $Å^{-1}$), with three replications for the simulation system.

The MM-GBSA method(Genheden and Ryde, 2015) was applied to estimate the binding free energy between hZnT1 and CDDO-ME. To achieve this aim, 100 snapshots were collected from the last 100 ns of the MD trajectory. The binding free energy was calculated by the formula:

$$\Delta G_{MMGBSA} = G_{compiex} - G_{receptor} - G_{ligand}$$

Real-time quantitative PCR (RT-qPCR)

Total intracellular RNA was extracted using EasyPure RNA Purification Kit (TransGen, China). A quantitative RT-qPCR assay was performed using a SYBR

Premix Green RT-PCR kit (Biorad, USA) following the manufacture's protocol. Amplification was carried out using a thermocycler (Roche, Switzerland). Primer sequences were: GAPDH forward primer 5′-CCCACTCCTCCACCTTTGACG-3′ and reverse primer 5′-CACCACCCTGTTGCTGTAGCCA-3′, hZnT1 forward primer 5′-ATACCAGCAACTCCAACGGG-3′ and reverse primer 5′-CTGGGGTTTTCTGGGTCTGC-3′. hZnT1 and GAPDH transcript levels were determined by the ΔΔCT method.

Cell viability of CDDO-ME

Cell viability of CDDO-ME was performed on Hela, sh-NC and sh-hZnT1 cell lines using a Cell Counting Kit-8 (CCK-8, Beyotime, China). Serial dilutions of the CDDO-ME (0.08-20 μM in DMEM) were added and incubated for 72 h at 37°C with 5% CO_2 in a 96 well plate. Cells were incubated for 1 h with 10 μL of CCK-8. The microplate reader was used to measure the absorbance at 450 nm. The viability of cells treated with CDDO-ME was relativized to that of the non-treated cells.

Cell colony formation assay

Hela, sh-NC and sh-hZnT1 cells were seeded into 6-well cell culture plates containing 10% FBS medium (1000 cells/well), and cultured at 37°C, 5% CO_2 incubator for 2 days. The cells were treated with a gradient concentration of CDDO-ME (0, 20 and 40 nM) for another 10 days. Cells were washed with phosphate-buffered saline (PBS) three times and fixed with 4% paraformaldehyde (PFA) for 20 min at room temperature. Cell colonies were stained with crystal violet for 15 min and washed with PBS three times. Finally, the cell plates were allowed to dry at room temperature and photographed. The number of cell colonies were counted and quantified.

Wound Healing Assay

Hela, sh-NC and sh-hZnT1 cells were plated on 6-well plates at a density of 1×10^6 cells/well. Next day, the cell monolayers were scratched in a single straight line using a 200 μL pipette tip. Subsequently, the cells were washed with PBS and treated with CDDO-ME (0-500 nM) for 24 h, 48 h and 72 h. The images were captured by an

inverted fluorescence microscope, and the wound recovery rates were measured using the following equation: Relative wound healing (%) = (wound area at Tt/wound area at T0) × 100. The wound area was measured by ImageJ.

Animal experiments

Animal experiments were done in accordance with the authorization of animal operation and in accordance with the China law for animal protection. This study and experimental protocol were approved by the Animal and Welfare Committee of Tianjin University (Approval number: TJUE-2024-053). All animal procedures described in this work were performed using 5-week-old female BALB/c nude mice, which were purchased from SPF Beijing SiPeiFu Biotechnology Co., Ltd., Beijing, China. 5×10^6 Hela cells were inoculated on the right back of BALB/c nude mice near the armpit. After 13 days, the mice were divided into 3 groups (n = 6 per group): (1) control group, (2) 5 mg/kg (17 μM) cisplatin group, (3) 10 mg/kg (20 μM) CDDO-ME group. Mice in the cisplatin and CDDO-ME groups were injected with 0.1 mL of working solution every 3 days, while control group mice were injected with 0.1 mL of PBS. The length and width of tumor volumes were measured every 3 days using a vernier caliper, and calculated according to the standard formula (length × width^2/2). The mice were weighed every three days. All the mice were sacrificed after 7 times injection from the beginning of the experiments. The tumors were stripped and weighed.

Statistical analysis

The graphical presentation and data analysis were conducted using GraphPad Prism 9. The data are displayed as mean ± Standard error of mean (SEM). Statistical significance of the differences between group means was evaluated by one-way analysis of variance (ANOVA) using the Tukey honestly significant difference test as a post hoc test; P values≤0.05 were considered statistically significant (*, $P < 0.05$; **, $P < 0.01$; ***, $P < 0.001$; ****, $P < 0.0001$, ns, not significant).

Supplementary Figures

Figure S1. Cryo-EM structure determination of hZnT1. (A) Size exclusion chromatography and SDS-PAGE analysis. (B) Representative micrographs and 2D class averages. (C) Cryo-EM workflow chart. (D) Gold-standard FSC curves. (E) Angular distribution calculated in cryoSPARC for hZnT1. The heat map shows the number of particles for each viewing angle (less = blue, more = red).

Figure S2. Cryo-EM density maps and interactions of hZnT1. (A) Cryo-EM density maps of TM1-6 and the CTD of hZnT1. Numbers indicate the TM helix and CTD number from the N-terminus. (B) The hydrophobic interactions of TM2 and TM3. (C) The overall interactions of dimeric hZnT1. The figure is generated by LigPlot+.

Figure S3. Cryo-EM structure determination of hZnT1-CDDO-ME. (A) Representative micrographs of data collection. (B) The 2D class averages. (C) Cryo-EM workflow chart. (D) Gold-standard FSC curves. (E) Angular distribution calculated in cryoSPARC for hZnT1-CDDO-ME. The heat map shows the number of particles for each viewing angle (less = blue, more = red).

Figure S4. Cryo-EM density maps of hZnT1-CDDO-ME. Cryo-EM density maps of of hZnT1-CDDO-ME. The TM1-6 and the CTD of hZnT1 are presented. Numbers indicate the TM helix and CTD number from the N-terminus.

Figure S5. Sequence alignments of hZnTs and YiiP. The secondary structure and His-rich loop of hZnT1 are indicated.

Figure S6. The structure of hZnT1-CDDO-ME and MD simulations. (A) The comparison of hZnT1-CDDO-ME (green and pink) and hZnT1-apo (white). The cryo-

EM density of H43 and E99 in hZnT1-CDDO-ME and hZnT1-apo are shown on the right, respectively. (B) The hydrophobic interactions between CDDO-ME and hZnT1. (C) The simulation system containing hZnT1, POPC, and CDDO-ME was generated by CHARMM-GUI. (D) The RMSD of hZnT1 protein. (E) The RMSF of hZnT1 protein. (F) The overall radius of gyration of hZnT1. (G) The RMSD of hZnT1 and CDDO-ME.

Figure S7. The binding affinities of WT and mutated hZnT1 with CDDO-ME.

Figure S8. Structure superimpositions of OF-hZnT1, IF-hZnT1 and CDDO-ME-hZnT1. (A) Structure superimposition of hZnT1 Zn^{2+} unbound OF homodimer (green) and hZnT1 Zn^{2+} bound IF homodimer (PDB: 8XMF, slate). (B) Structure superimposition of hZnT1 Zn^{2+} bound IF homodimer (PDB: 8XMF, slate) and CDDO-ME-hZnT1 (pink). (C) CDDO-ME occupies the Zn^{2+} cavity and sterically hinders the movement of TM2. (D) The conformational changes of OF-hZnT1 (green) TMs compared with IF-hZnT1 (slate). (E) The conformational changes of CDDO-ME-hZnT1 (pink) TMs compared with IF-hZnT1 (slate).

Supplementary Tables:

Table S1. Cryo-EM data collection, refinement, and validation statistics

Table S2. The hydrogen bonds of hZnT1 protomers.

Table S3. Virtual screening based on a marketed drug library.

Figure S1

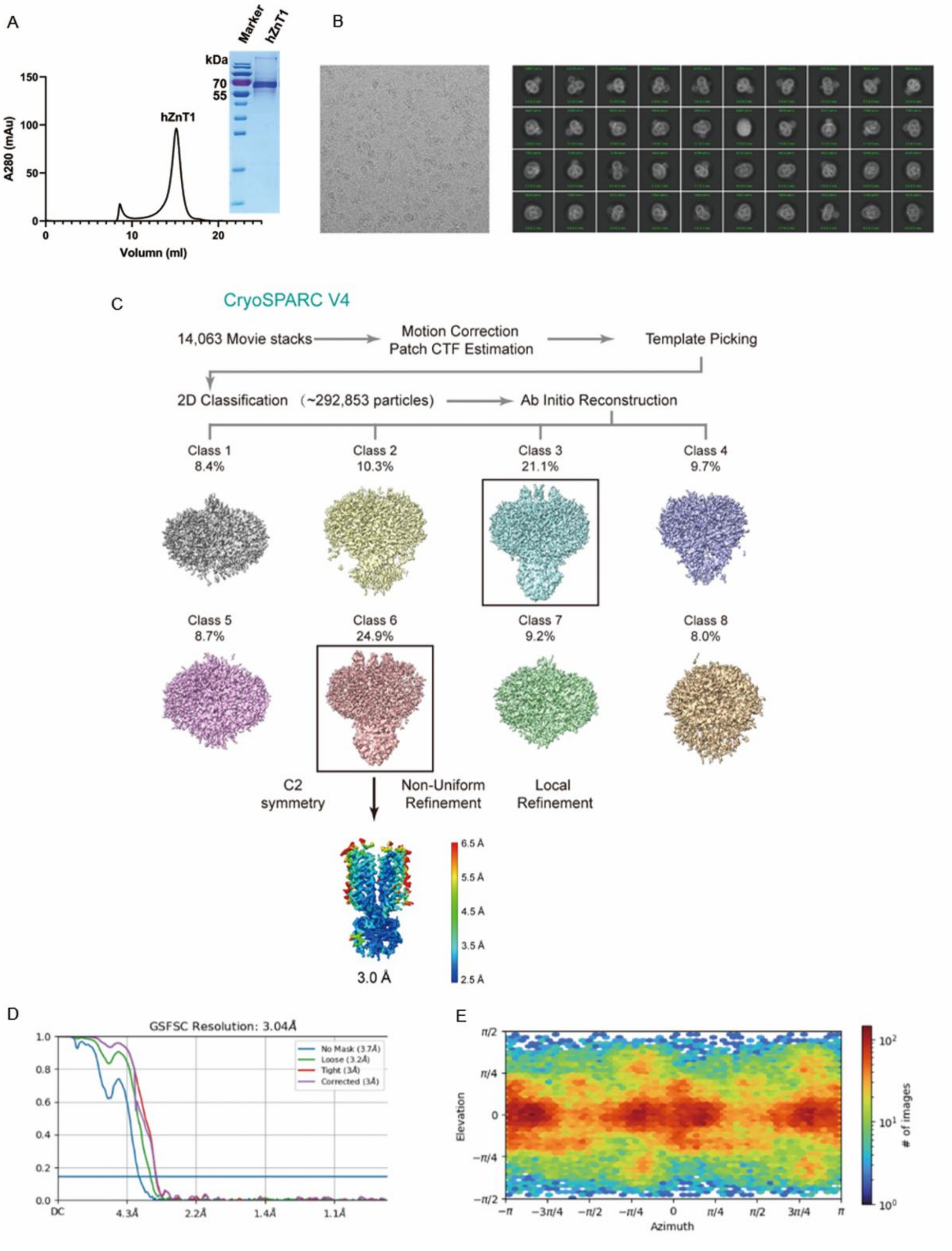
A
Marker
hZnT1
kDa
70
55
hZnT1
A280 (mAu)
Volumn (ml)
B
C
CryoSPARC V4
14,063 Movie stacks
Motion Correction
Patch CTF Estimation
Template Picking
2D Classification (~292,853 particles)
Ab Initio Reconstruction
Class 1 8.4%
Class 2 10.3%
Class 3 21.1%
Class 4 9.7%
Class 5 8.7%
Class 6 24.9%
Class 7 9.2%
Class 8 8.0%
C2 symmetry
Non-Uniform Refinement
Local Refinement
3.0 Å
D
GSFSC Resolution: 3.04Å
No Mask (3.7Å)
Loose (3.2Å)
Tight (3Å)
Corrected (3Å)
E
Elevation
Azimuth
# of images

Figure S2

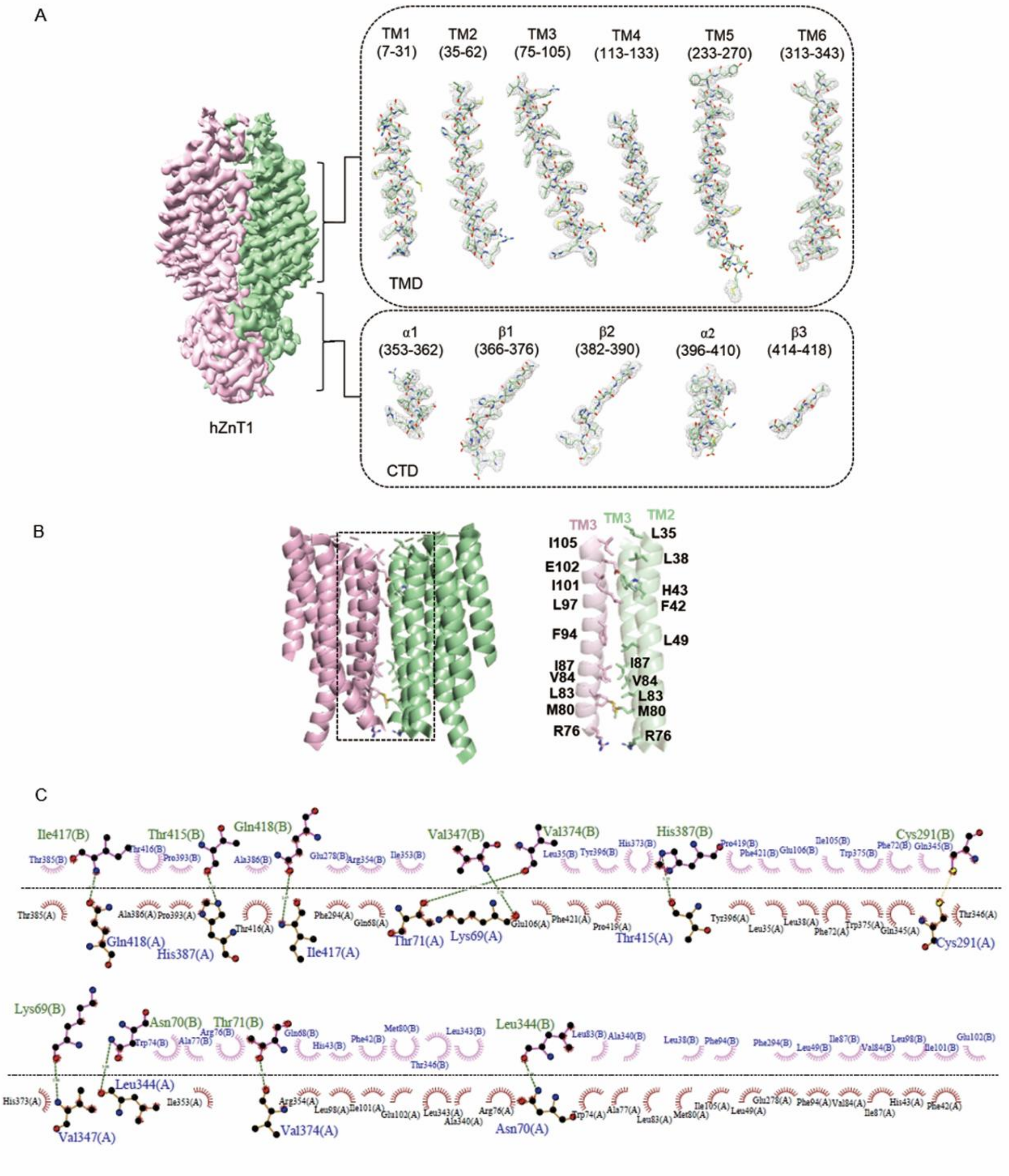
A
TM1
(7-31)
TM2
(35-62)
TM3
(75-105)
TM4
(113-133)
TM5
(233-270)
TM6
(313-343)
TMD
α1
(353-362)
β1
(366-376)
β2
(382-390)
α2
(396-410)
β3
(414-418)
CTD
hZnT1
B
TM3
TM3
TM2
I105
E102
I101
L97
F94
I87
V84
L83
M80
R76
L35
L38
H43
F42
L49
I87
V84
L83
M80
R76
C
Ile417(B)
Thr415(B)
Gln418(B)
Val347(B)
Val374(B)
His387(B)
Cys291(B)
Gln418(A)
His387(A)
Ile417(A)
Thr71(A)
Lys69(A)
Thr415(A)
Cys291(A)
Lys69(B)
Asn70(B)
Thr71(B)
Leu344(B)
Leu344(A)
Val347(A)
Val374(A)
Asn70(A)

Figure S3

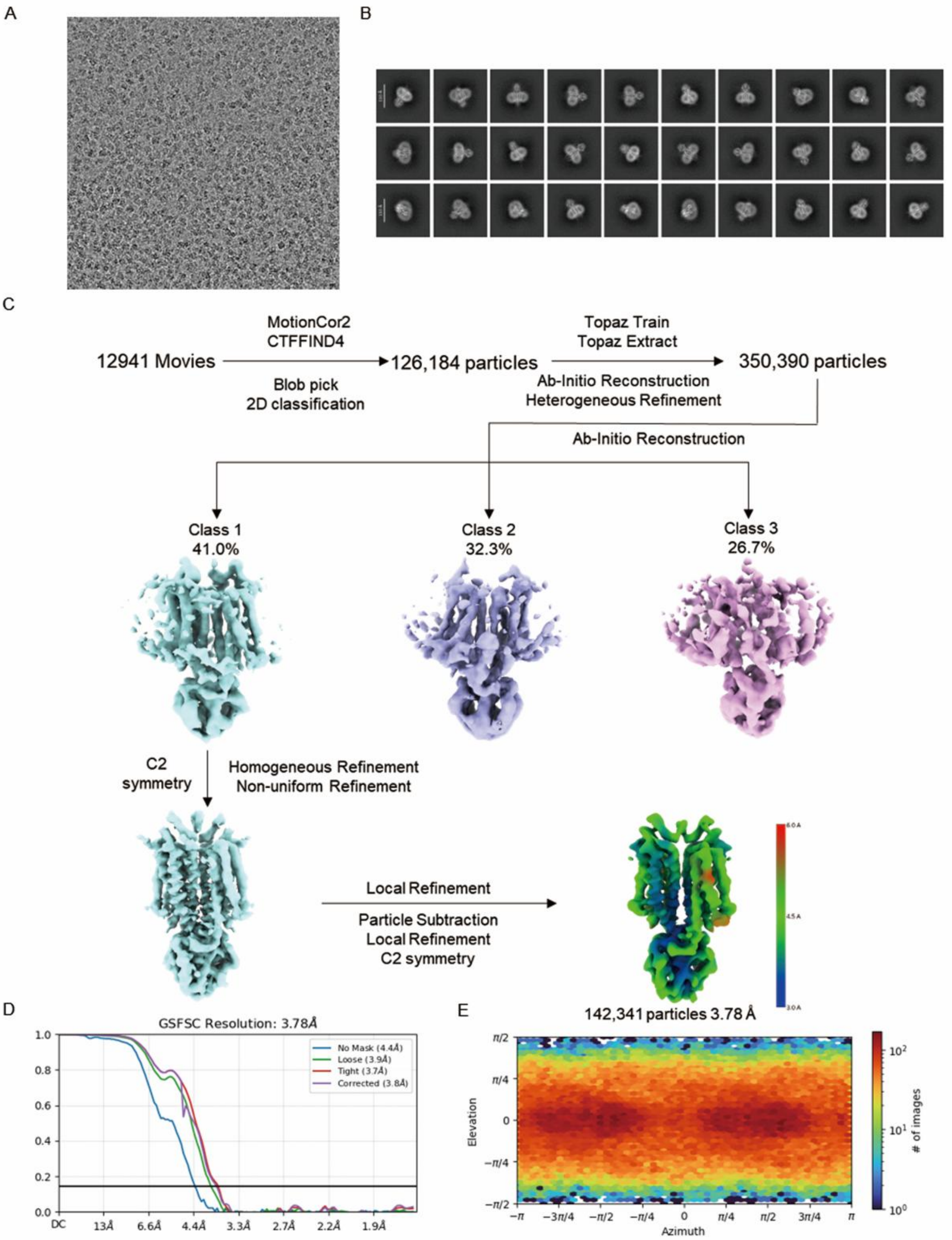
A
B
C
12941 Movies
MotionCor2
CTFFIND4
Blob pick
2D classification
126,184 particles
Topaz Train
Topaz Extract
Ab-Initio Reconstruction
Heterogeneous Refinement
350,390 particles
Ab-Initio Reconstruction
Class 1
41.0%
Class 2
32.3%
Class 3
26.7%
C2
symmetry
Homogeneous Refinement
Non-uniform Refinement
Local Refinement
Particle Subtraction
Local Refinement
C2 symmetry
6.0 Å
4.5 Å
3.0 Å
D
GSFSC Resolution: 3.78Å
No Mask (4.4Å)
Loose (3.9Å)
Tight (3.7Å)
Corrected (3.8Å)
1.0
0.8
0.6
0.4
0.2
0.0
DC
13Å
6.6Å
4.4Å
3.3Å
2.7Å
2.2Å
1.9Å
E
142,341 particles 3.78 Å
Elevation
Azimuth
# of images

Figure S4

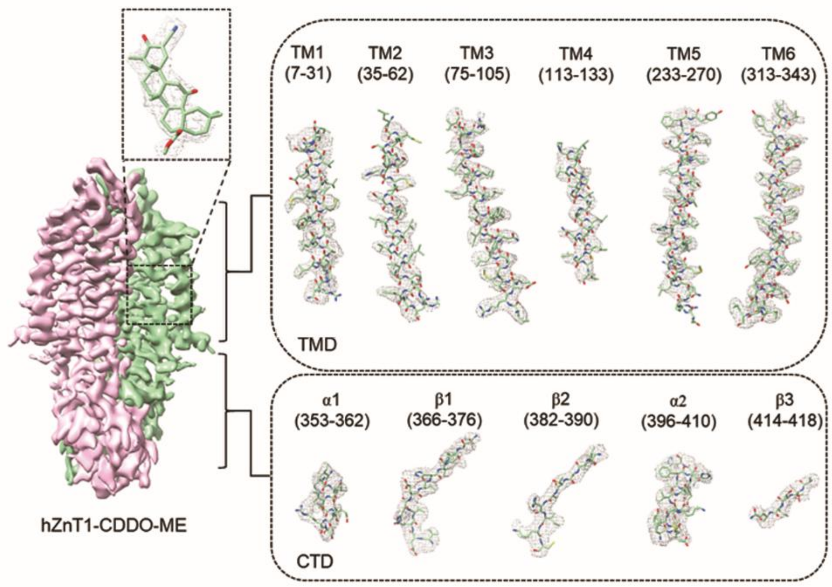

TM1
(7-31)
TM2
(35-62)
TM3
(75-105)
TM4
(113-133)
TM5
(233-270)
TM6
(313-343)
TMD
α1
(353-362)
β1
(366-376)
β2
(382-390)
α2
(396-410)
β3
(414-418)
CTD
hZnT1-CDDO-ME

Figure S5

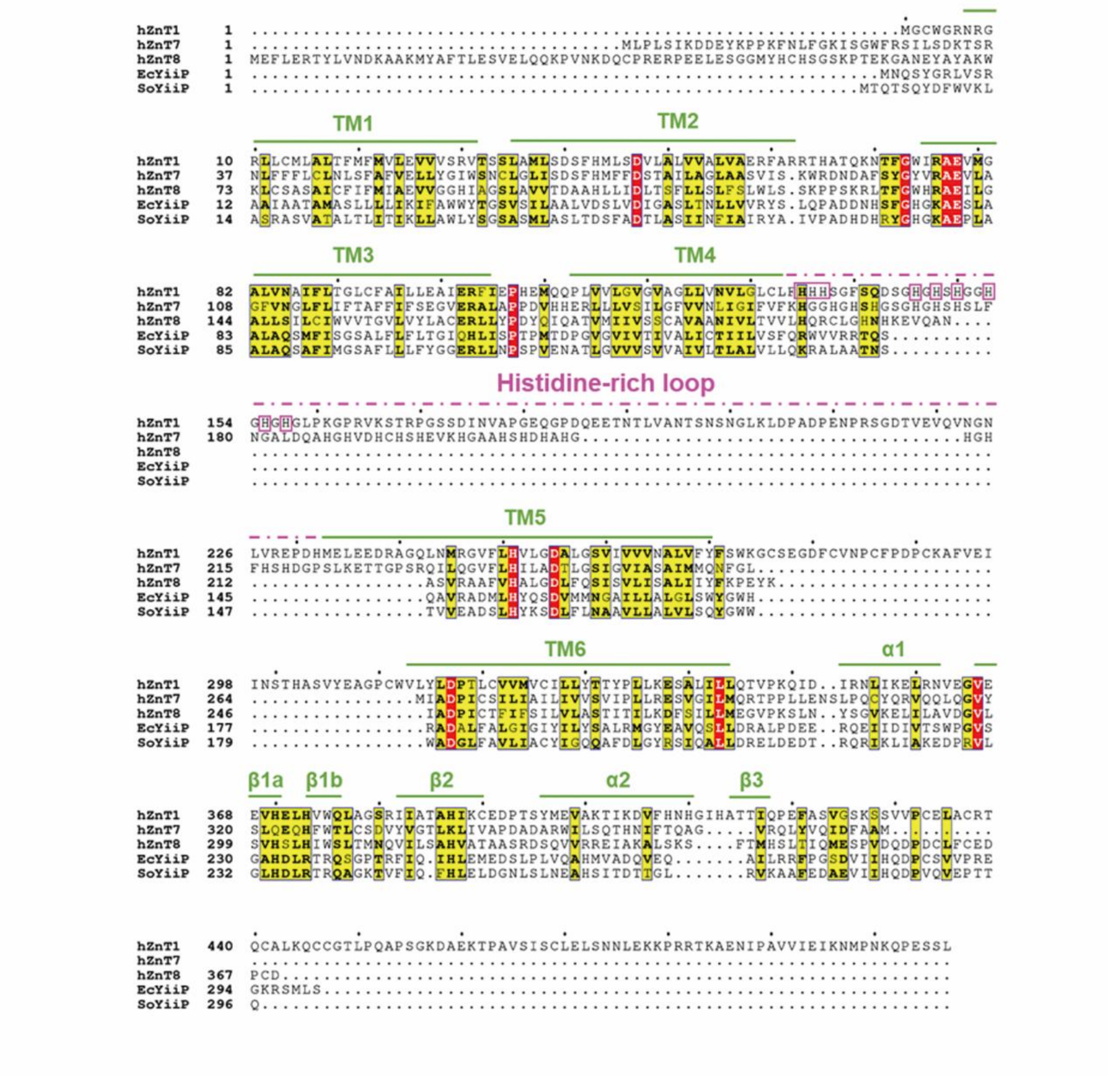

TM1
TM2
TM3
TM4
Histidine-rich loop
TM5
TM6
α1
β1a
β1b
β2
α2
β3
hZnT1
hZnT7
hZnT8
EcYiiP
SoYiiP

Figure S6

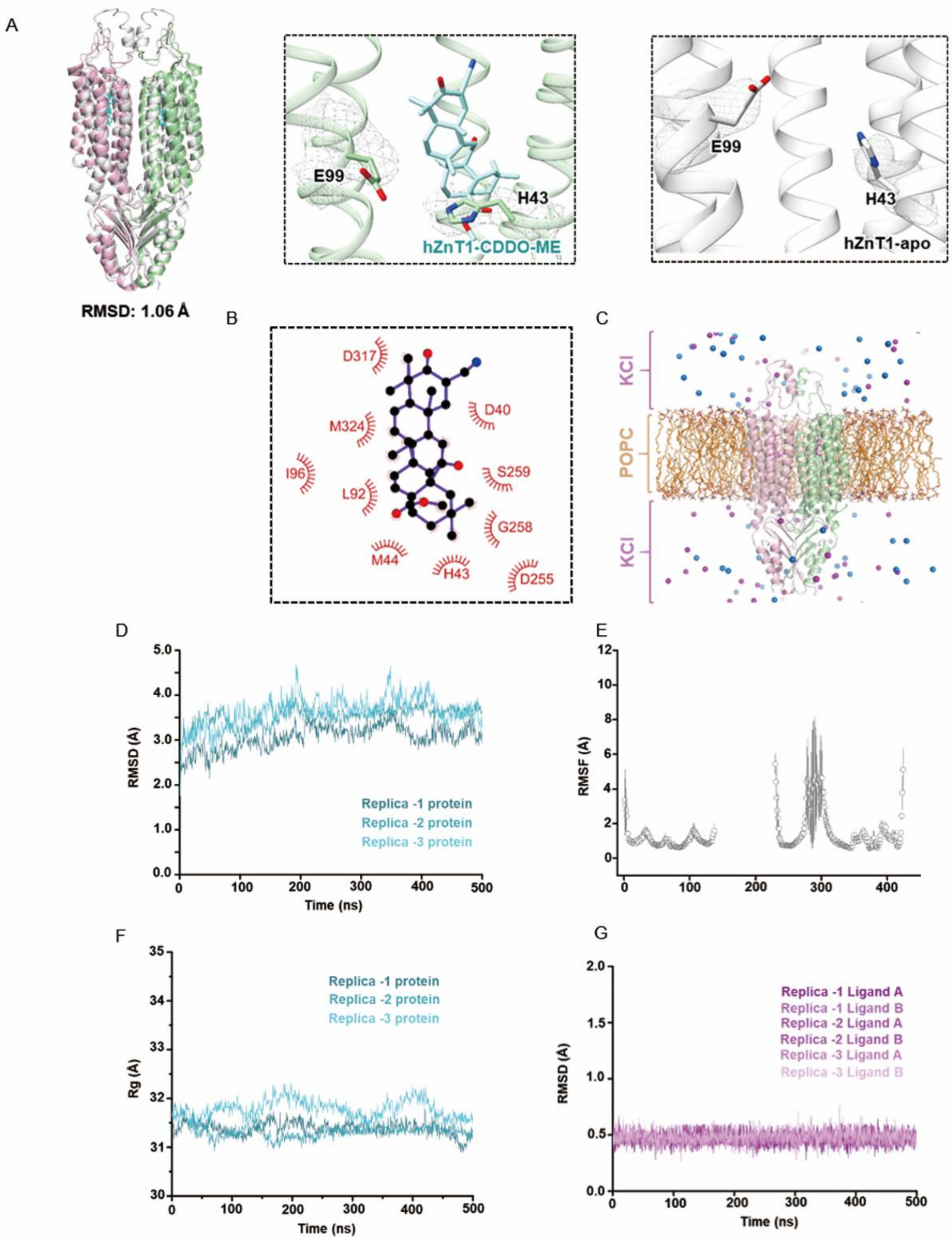
A
E99
H43
hZnT1-CDDO-ME
E99
H43
hZnT1-apo
RMSD: 1.06 Å
B
D317
D40
M324
I96
S259
L92
G258
M44
H43
D255
C
KCl
POPC
KCl
D
RMSD (Å)
Time (ns)
Replica -1 protein
Replica -2 protein
Replica -3 protein
E
RMSF (Å)
F
Rg (Å)
Time (ns)
Replica -1 protein
Replica -2 protein
Replica -3 protein
G
RMSD (Å)
Time (ns)
Replica -1 Ligand A
Replica -1 Ligand B
Replica -2 Ligand A
Replica -2 Ligand B
Replica -3 Ligand A
Replica -3 Ligand B

Figure S7

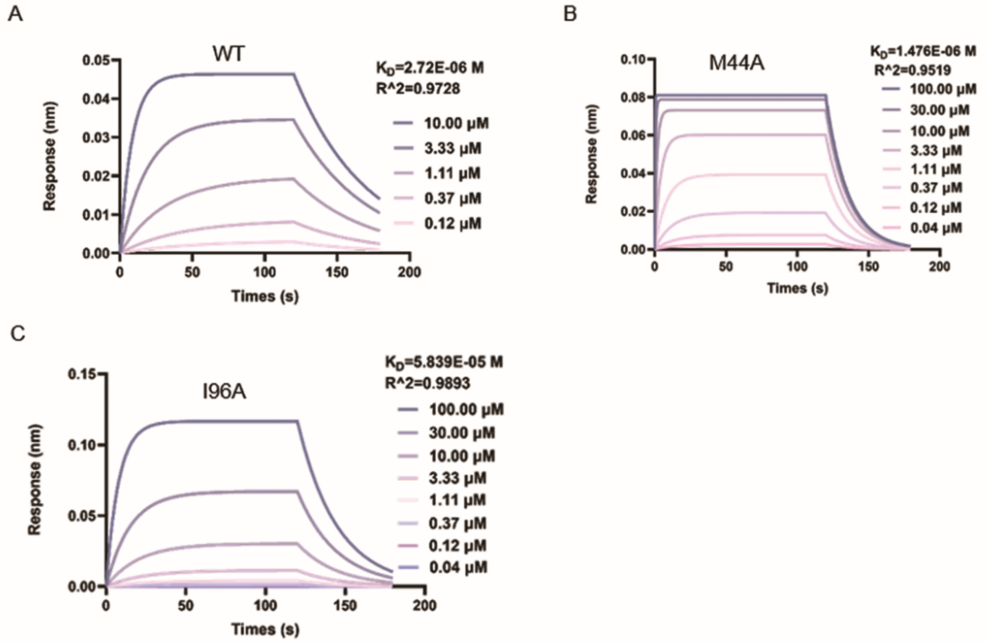

A
WT
K_D=2.72E-06 M
R^2=0.9728
10.00 µM
3.33 µM
1.11 µM
0.37 µM
0.12 µM
Response (nm)
Times (s)
B
M44A
K_D=1.476E-06 M
R^2=0.9519
100.00 µM
30.00 µM
10.00 µM
3.33 µM
1.11 µM
0.37 µM
0.12 µM
0.04 µM
Response (nm)
Times (s)
C
I96A
K_D=5.839E-05 M
R^2=0.9893
100.00 µM
30.00 µM
10.00 µM
3.33 µM
1.11 µM
0.37 µM
0.12 µM
0.04 µM
Response (nm)
Times (s)

Figure S8

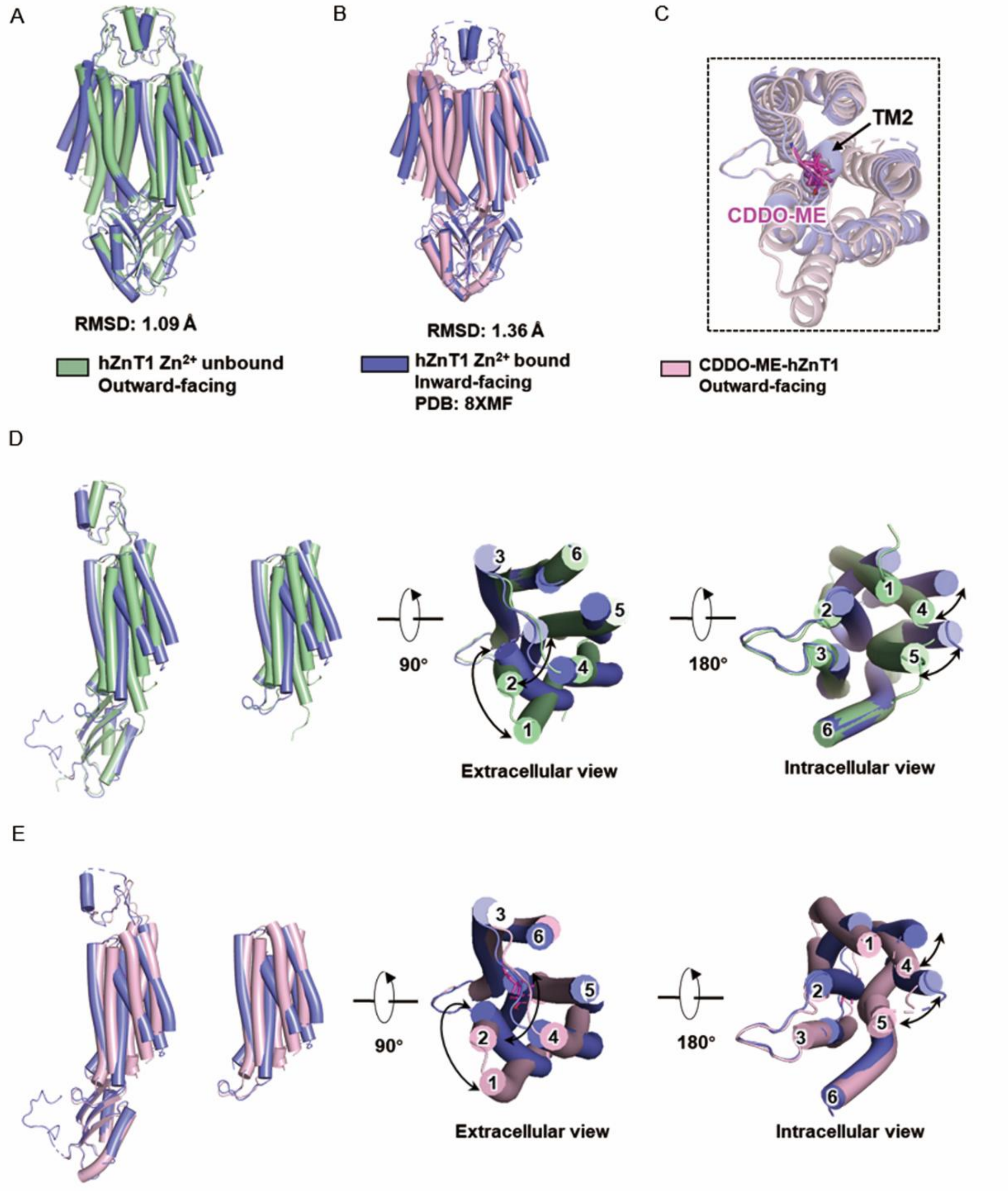

259
260

Table S1. Cryo-EM data collection, refinement, and validation statistics.

	hZNT1 (PDB: 9KZW)	hZnT1-CDDO-ME (PDB: 9L00)
**Data collection and processing**		
Microscope	FEI Titan Krios	Thermo Fisher Titan Krios
Magnification	270,000	96,000
Voltage (KV)	300	300
Detector	Gatan K3	Falcon 4
Electron exposure (e^-/ Å^2)	50	50
Defocus range (μm)	-0.5 to -1.2	-1.0 to -2.0
Pixel size (Å)	0.45	0.83
Symmetry imposed	C2	C2
Initial particle images (no.)	568,835	350,390
Final particle images (no.)	292,853	142,341
Map resolution (Å)	3.04	3.78
FSC threshold	0.143	0.143
**Refinement**		
Initial model used (PDB code)	6XPE	9KZW
Model resolution (Å)	3.0	3.78
FSC threshold	0.143	0.143
Map sharpening *B* factor (Å^2)	87.9	177.2
Model composition		
Non-hydrogen atoms	5226	4854
Protein residues	668	614
Water	/	/
Ligands	0	2
B factors		
Protein	70.77	81.02
Ligand	/	87.20
Water	/	/
R.m.s. deviations		
Bond lengths (Å)	0.003	0.004
Bond angles (°)	0.525	0.723
Valiation		
MolProbity score	1.77	2.55
Clash score	9.22	9.96
Rotamer outliers (%)	0.00	0.00
Ramachandran plot		
Favored (%)	95.91	92.19
Allowed (%)	4.09	7.81
Outliers (%)	0.00	0.00

Table S2. The hydrogen bonds of hZnT1 protomers.

Chain B	Dist. [Å]	Chain A
VAL 347 [N]	2.78	LYS 69 [O]
ASN 70 [ND2]	3.08	LEU 344 [O]
THR 71 [OG1]	3.16	VAL 374 [O]
HIS 387 [NE2]	3.25	THR 415 [O]
ILE 417 [N]	2.98	GLN 418 [OE1]
LYS 69 [O]	2.80	VAL 347 [N]
LEU 344 [O]	3.08	ASN 70 [ND2]
VAL 374 [O]	3.15	THR 71 [OG1]
THR 415 [O]	3.27	HIS 387 [NE2]
GLN 418 [OE1]	2.97	ILE 417 [N]

Table S3. Virtual screening based on a marketed drug library.

Name	Affinity (kcal/mol)	CAS number
Rupatadine Fumarate	-10	182349-12-8
Bardoxolone methyl	-10	218600-53-4
Larotrectinib	-10.1	1223403-58-4
Rimegepant	-10.8	1289023-67-1
Bictegravir	-10.1	1611493-60-7
Limonin	-10.4	1180-71-8
Etoposide	-10.3	33419-42-0
Mizolastine	-10	108612-45-9
Paliperidone	-10	144598-75-4
Capmatinib	-10.6	1029714-89-3

SI References

Adams, P.D., Afonine, P.V., Bunkoczi, G., Chen, V.B., Davis, I.W., Echols, N., Headd, J.J., Hung, L.W., Kapral, G.J., Grosse-Kunstleve, R.W., *et al.* (2010). PHENIX: a comprehensive Python-based system for macromolecular structure solution. Acta Crystallogr D Biol Crystallogr 66, 213-221.

Cheatham, T.E., and Joung, I.S. (2009). Molecular Dynamics Simulations of the Dynamic and Energetic Properties of Alkali and Halide Ions Using Water-Model-Specific Ion Parameters. Journal of Physical Chemistry B 113, 13279-13290.

Chen, V.B., Arendall, W.B., 3rd, Headd, J.J., Keedy, D.A., Immormino, R.M., Kapral, G.J., Murray, L.W., Richardson, J.S., and Richardson, D.C. (2010). MolProbity: all-atom structure validation for macromolecular crystallography. Acta Crystallogr D Biol Crystallogr 66, 12-21.

D.A. Case, H.M.A., K. Belfon, I.Y. Ben-Shalom, J.T. Berryman, S.R. Brozell, D.S. Cerutti, T.E. Cheatham, III, G.A. Cisneros, V.W.D. Cruzeiro, T.A. Darden, R.E. Duke, G. Giambasu, M.K. Gilson, H. Gohlke, A.W. Goetz, R. Harris, S. Izadi, S.A. Izmailov, K. Kasavajhala, M.C. Kaymak, E. King, A. Kovalenko, T. Kurtzman, T.S. Lee, S. LeGrand, P. Li, C. Lin, J. Liu, T. Luchko, R. Luo, M. Machado, V. Man, M. Manathunga, K.M. Merz, Y. Miao, O. Mikhailovskii, G. Monard, H. Nguyen, K.A. O'Hearn, A. Onufriev, F. Pan, S. Pantano, R. Qi, A. Rahnamoun, D.R. Roe, A. Roitberg, C. Sagui, S. Schott-Verdugo, A. Shajan, J. Shen, C.L. Simmerling, N.R. Skrynnikov, J. Smith, J. Swails, R.C. Walker, J. Wang, J. Wang, H. Wei, R.M. Wolf, X. Wu, Y. Xiong, Y. Xue, D.M. York, S. Zhao, and P.A. Kollman (2022). Amber 2022, University of California, San Francisco.

Emsley, P., Lohkamp, B., Scott, W.G., and Cowtan, K. (2010). Features and development of Coot. Acta Crystallogr D Biol Crystallogr 66, 486-501.

Genheden, S., and Ryde, U. (2015). The MM/PBSA and MM/GBSA methods to estimate ligand-binding affinities. Expert Opin Drug Discov 10, 449-461.

Long, Y., Zhu, Z., Zhou, Z., Yang, C., Chao, Y., Wang, Y., Zhou, Q., Wang, M.W., and Qu, Q. (2024). Structural insights into human zinc transporter ZnT1 mediated Zn(2+) efflux. EMBO Rep 25, 5006-5025.

Pettersen, E.F., Goddard, T.D., Huang, C.C., Couch, G.S., Greenblatt, D.M., Meng, E.C., and Ferrin, T.E. (2004). UCSF Chimera--a visualization system for exploratory research and analysis. J Comput Chem 25, 1605-1612.

Punjani, A., Rubinstein, J.L., Fleet, D.J., and Brubaker, M.A. (2017). cryoSPARC: algorithms for rapid unsupervised cryo-EM structure determination. Nat Methods 14, 290-296.

Rohou, A., and Grigorieff, N. (2015). CTFFIND4: Fast and accurate defocus estimation from electron micrographs. J Struct Biol 192, 216-221.

Shi, S., Ma, B., Ji, Q., Guo, S., An, H., and Ye, S. (2023). Identification of a druggable pocket of the calcium-activated chloride channel TMEM16A in its open state. J Biol Chem, 104780.

Trott, O., and Olson, A.J. (2010). AutoDock Vina: improving the speed and accuracy of docking with a new scoring function, efficient optimization, and multithreading. J Comput Chem 31, 455-461.
Xue, J., Xie, T., Zeng, W., Jiang, Y., and Bai, X.C. (2020). Cryo-EM structures of human ZnT8 in both outward- and inward-facing conformations. Elife 9.
